# Repurposing pexmetinib as an inhibitor of TKI-resistant BCR::ABL1

**DOI:** 10.1038/s41375-024-02282-y

**Published:** 2024-05-15

**Authors:** Diletta Fontana, Federica Malighetti, Matteo Villa, Alfonso Zambon, Carlo Gambacorti-Passerini, Luca Mologni

**Affiliations:** 1 Department of Medicine and Surgery, University of Milano-Bicocca, Monza, Italy; 2 Department of Chemistry and Geological Sciences, University of Modena and Reggio Emilia, Modena, Italy; 3 Department of Hematology, Fondazione IRCCS San Gerardo dei Tintori, Monza, Italy

**Keywords:** Drug development, Haematological cancer

## To the Editor:

Chronic myeloid leukemia (CML) is a clonal disorder caused by the Philadelphia (Ph) chromosome encoding for the *BCR::ABL1* fusion gene [1]. The development of imatinib and other tyrosine kinase inhibitors (TKIs) targeting the ABL1 tyrosine kinase allowed a dramatic change in CML prognosis, with long-term control of the disease in most individuals [2]. In spite of excellent results, a non-negligible fraction of patients fails to achieve a complete remission or develops resistance to TKIs due to the acquisition of point mutations that affect drug binding to the active site of the kinase [3]. The T315I substitution at the gatekeeper site is the most intractable BCR::ABL1 mutant, being highly resistant to most approved inhibitors except ponatinib, which is associated with serious cardiovascular adverse events [4], and the non-ATP competitive compound asciminib [5]. Similar to CML, Ph+ acute lymphoblastic leukemia is characterized by expression of the *BCR::ABL1* fusion oncogene. Consequently, the use of imatinib and other TKIs brought clinical improvement to the patients [6]. In this setting, as well, relapses are frequent due to the development of resistant disease, highlighting the need to develop new treatments. Again, the T315I mutant is the major drug-resistant clone observed in relapsing patients. Considering the number of CML patients who still need to switch/suspend therapy due to any reason, be it resistance or intolerance, the identification of new agents against the disease may be beneficial. In this work, we identified pexmetinib (ARRY-614) as an inhibitor of drug-resistant BCR::ABL1, including G250E, Y253F, E255K/V and T315I mutants.

**Fig. 1:**
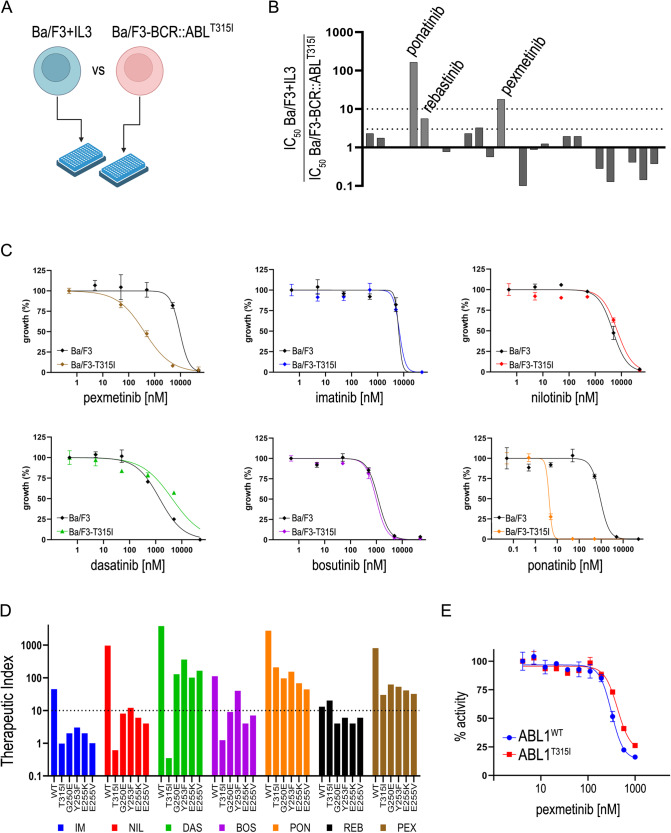
Identification of pexmetinib activity in Ba/F3^T315I^ cells. **A** Scheme of the screening strategy. The sensitivity of Ba/F3 and Ba/F3^T315I^ cells to 10 µM inhibitors was assessed in 96-well plates. **B** IC_50_ ratio (parental/T315I) of 27 inhibitors that passed phase 1 (listed in Supplementary Fig. 1); the indicated three compounds were selected after phase 2 of the screening. **C** Ba/F3 cells, parental or expressing BCR::ABL1^T315I^ mutant, were treated with increasing concentrations of the indicated inhibitors. Dose-response curves were obtained by non-linear fitting of normalized cell growth data. **D** A summary of the experimental therapeutic indexes (calculated as the [Ba/F3^IL3^]/[Ba/F3^BCR::ABL1^] IC_50_ ratio) is reported for WT and mutant cells (T315I, G250E, Y253F, E255K, E255V); pexmetinib and ponatinib pass the tenfold therapeutic index mark for all mutations, indicating they can safely be used against these mutants, without killing non-transformed cells. IM imatinib; NIL nilotinib; DAS dasatinib; BOS bosutinib; PON ponatinib; REB rebastinib; PEX pexmetinib. **E** Inhibition of recombinant WT and T315I ABL1 kinase by pexmetinib in an in vitro kinase assay.

We screened a kinase-focused compound library for selective inhibition of ABL1^T315I^ in cells, using an isogenic Ba/F3 cellular model (Fig. 1A and Supplementary Fig. 1). Ba/F3 cells expressing the mutant *BCR::ABL1-T315I* oncogene (Ba/F3^T315I^) were obtained by electroporation as previously described [7]. Specific cytotoxicity for Ba/F3^T315I^ versus parental interleukin 3 (IL-3)-dependent Ba/F3 cells was sought by MTS viability assays. First, 627 compounds were tested at 10 µM concentration against the two cell lines: a selectivity (i.e., the ratio between Ba/F3 + IL-3 and Ba/F3^T315I^ viability) threshold of 5 was arbitrarily set. From the whole library, 27 compounds survived this filter and were passed onto the next stage (Supplementary Fig. 2). Here, dose-response curves were generated using the candidate compounds against parental Ba/F3 and Ba/F3^T315I^ cells. In this phase, IC_50_ values were used to rank the compounds. Only three drugs were finally selected by the screening, having an IC_50_ selectivity >3 versus parental cells (Fig. 1B). Two of them (ponatinib and rebastinib) are known ABL1^T315I^ inhibitors [7]. The third compound passing the filters, pexmetinib (ARRY-614), has been described as a dual Tie2 and p38^MAPK^ inhibitor with efficacy in preclinical models of leukemia [8]. The compound has off-target activity against wild-type (WT) ABL1 kinase.

Pexmetinib was thus selected for further characterization. The compound was compared to five clinical ABL1 inhibitors (imatinib, nilotinib, dasatinib, bosutinib and ponatinib) and was the only one to show specific cytotoxic activity against Ba/F3^T315I^ cells, except ponatinib (Fig. 1C). When tested on Ba/F3 cells expressing the WT BCR::ABL1 fusion, pexmetinib showed comparable activity to bosutinib and nilotinib (Supplementary Fig. 3A). Interestingly, pexmetinib was the least toxic drug on parental Ba/F3 cells driven by IL-3. Owing to this, ponatinib and pexmetinib were the only two compounds with >10-fold selectivity for all tested ABL1 mutants versus parental Ba/F3 cells (Fig. 1D and Supplementary Tables 1, 2), i.e., they had a safe therapeutic index, defined as the ratio between undesired activity on Ba/F3 cells and specific activity on kinase-addicted target cells [9]. These results suggested that pexmetinib has specific anti-ABL1^T315I^ activity in an experimental cell model.

In biochemical assays, pexmetinib showed comparable activity on the purified recombinant ABL1^WT^ and ABL1^T315I^ enzymes (Fig. 1E), again confirming a minor loss of potency in the presence of the gatekeeper mutation.

**Fig. 2:**
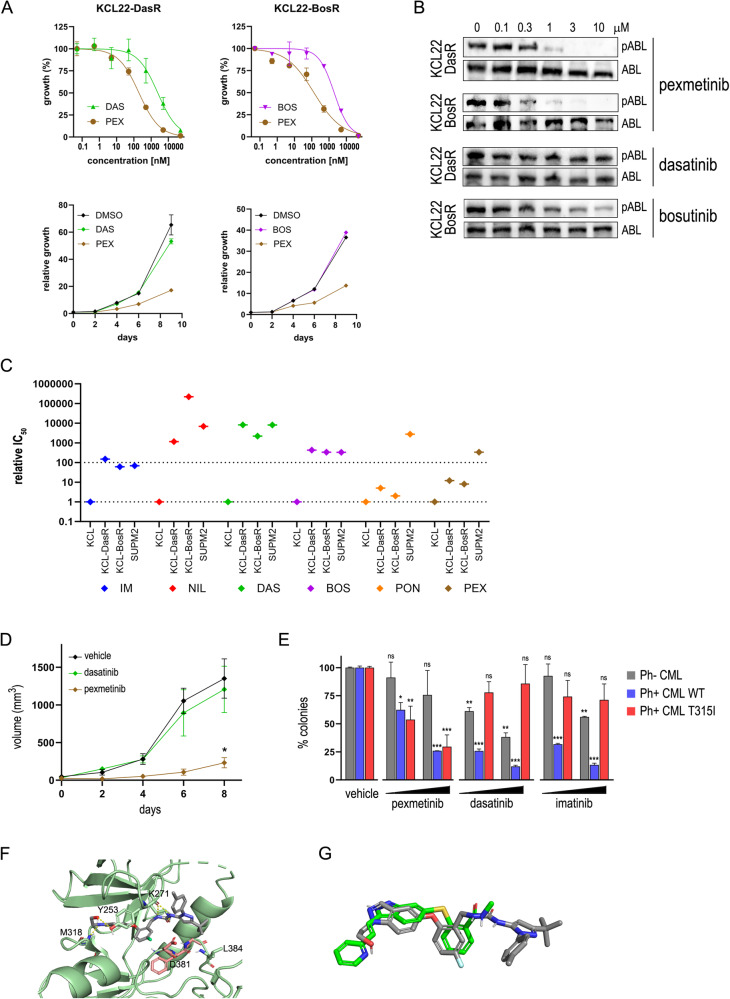
Effects of pexmetinib on human CML cells. **A** Proliferation of human Ph+ KCL22 cells carrying the T315I mutation treated with the indicated inhibitors. Dose-response curves (upper panels) and time-course experiments (lower panels) are shown. KCL22-DasR and KCL22-BosR indicate KCL22 cells selected for resistance to dasatinib and bosutinib, respectively. **B** Inhibition of BCR::ABL1^T315I^ autophosphorylation in drug-resistant KCL22 cells, shown by western blot using phospho-specific (pABL1) and total ABL1 antibodies. **C** Summary of drug-resistant KCL22 cells growth inhibition data, shown as IC_50_ fold-change values relative to parental KCL22, highlighting the loss of potency in resistant cells; Ph− cells (SUPM2) are shown as a reference of unspecific toxicity. **D** Tumor growth of KCL-DasR xenografts in nude mice treated daily with vehicle (*n* = 6), dasatinib (20 mg/kg p.o.; *n* = 5), or pexmetinib (40 mg/kg i.p.; *n* = 5). *p < 0.05 versus vehicle and dasatinib. Mice were injected subcutaneously with 8 × 10^6^ cells in right flank; treatments started when tumors were measurable (30 mm^3^). **E** Inhibition of colony formation of BCR::ABL1-negative, WT and T315I patient-derived bone marrow myeloid cells by pexmetinib (3–10 μM), dasatinib (3–10 nM) and imatinib (0.3–1 μM); 2 × 10^5^ cells were seeded in methylcellulose and colonies were counted after 14 days. The data represent the normalized mean ± SD of triplicate experiments. The average of two T315I patients is reported. *p < 0.05; **p < 0.01; ***p < 0.001 versus vehicle. Statistical significance is calculated by unpaired, two-tailed Student’s *t*-test. **F** Molecular docking of pexmetinib (gray sticks) in T315I-mutated ABL1 kinase shown by ribbon representation. The DFG motif is in pink color. **G** Superposition of pexmetinib (gray) and axitinib (green) bound to ABL1^T315I^ kinase. Docking studies were performed using the Maestro suite from Schrodinger. IM imatinib; NIL nilotinib; DAS dasatinib; BOS bosutinib; PON ponatinib; PEX pexmetinib.

We then tested pexmetinib on human Ph+ CML cell lines. The compound showed cell growth inhibition of two KCL22-derived cell lines that were selected for resistance to dasatinib and bosutinib, both carrying the T315I substitution, named KCL22-DasR and KCL22-BosR, respectively (Fig. 2A and Supplementary Fig. 3B). Pexmetinib inhibited BCR::ABL1 autophosphorylation in KCL22-DasR and KCL22-BosR cells, whereas dasatinib and bosutinib were inactive up to 10 µM (Fig. 2B). While first- and second-generation TKIs suffered >100-fold IC_50_ shift in resistant compared to parental drug-sensitive KCL22 cells, with values close to BCR::ABL1-negative SUPM2 cells (a proxy of unspecific toxicity), ponatinib and pexmetinib showed a limited loss of activity on the T315I mutant, still far from the toxic doses needed to kill SUPM2 cells (Fig. 2C). In this setting, pexmetinib ranked as the second-best inhibitor after ponatinib. Next, in vivo efficacy of pexmetinib was tested using a xenograft model of KCL22-DasR cells in immunodeficient mice. While tumors were resistant to dasatinib, as expected, pexmetinib profoundly impaired tumor growth (Fig. 2D). We conclude that pexmetinib has in vivo therapeutic activity against drug-resistant CML.

Finally, activity on patients’ samples was evaluated. Bone marrow cells were obtained from a Ph-negative MDS/MPN with neutrophilia, a Ph+ CML carrying the native fusion protein, and two Ph+ T315I-mutated drug-resistant CML patients; the cells were seeded in semisolid methylcellulose in the presence of TKIs to assess compounds performance on clinical samples. The patients expressing the T315I mutation had a history of imatinib, nilotinib and dasatinib therapy failure. Pexmetinib showed dose-dependent inhibition of colony formation both in WT and mutated Ph+ CML patients without affecting Ph− cells, while dasatinib and imatinib were ineffective on T315I myeloid cells at clinically relevant doses, although they suppressed colonies growth from the WT patients (Fig. 2E). These data confirm that pexmetinib can be safely used to block the BCR::ABL1^T315I^ mutant, including primary samples from CML patients.

To identify a possible structural basis for the inhibition of mutant ABL1 by pexmetinib, we carried out docking studies on several co-crystal structures of ABL1^T315I^. Intriguingly, pexmetinib fails to yield meaningful poses when docked on co-crystal structures of inactive (DFG-out) ABL1^T315I^ with structurally analog type II inhibitors (PDB codes: 3OY3, 3KF4, 3QRJ). We thus docked pexmetinib on the co-crystal structure of axitinib with ABL1^T315I^ (4TWP) [10]. Axitinib has a peculiar type I mode of action, binding ABL1^T315I^ in the active (DFG-in) conformation. Docking of pexmetinib on 4TWP suggests a similar binding mode (Fig. 2F, G): pexmetinib binds to the active conformation of ABL1^T315I^ forming a series of H-bonds between the indole ring and Met318 of the hinge region, its hydroxyethyl substituent and the hydroxyl group of Tyr253, and the urea group to the backbone of Tyr253; in addition, the fluorophenyl and indole rings of pexmetinib interact with the phenyl ring of Tyr253 by pi-stacking. The carbonyl of the urea forms a further H-bond with Lys271 of the salt bridge. Finally, the pyrazole substituents of the inhibitor elaborate toward the P-loop with the tolyl group and toward the A-loop with the tert-butyl group, the latter forming van der Waals interactions with Leu384. As Tyr253 is predicted to be involved in pexmetinib binding, it is somewhat unexpected that the Y253F mutant appears similarly sensitive to the other mutants assessed in our Ba/F3 model. We hypothesize that bonding with the hydroxyl of Tyr253 only provides a fraction of the total binding energy, which is ensured by several additional interactions. Indeed, the Y253F mutant displays some reduction of sensitivity compared to the WT enzyme (Supplementary Tables 1, 2), indicating that loss of this H-bond partially affects drug binding. Surprisingly, a different mutation (Y253H) conferred nearly tenfold higher resistance to pexmetinib (IC_50_ = 2099 nM; therapeutic index = 7; Supplementary Fig. 4), confirming that position 253 is an important point of contact with the compound, and suggesting that Y253H might develop as a resistance mechanism under pexmetinib. To explain the different behavior of pexmetinib against ABL1^Y253F^ and ABL1^Y253H^, we docked the compound on the two mutated models of the 4TWP structure. Docking outputs confirmed that the missing H-bond with the Tyr hydroxyl group causes reduced affinity to both mutants; in addition, for the Y253H mutant, a less efficient arrangement of the pi-stacking with the inhibitor’s aromatic rings further lowers binding strength (Supplementary Fig. 5).

We report here preclinical proof of concept for pexmetinib repurposing as a novel inhibitor of the highly resistant BCR::ABL1^T315I^ mutant, as well as of other imatinib-resistant mutations that still require careful selection of second-line therapy [11]. The gatekeeper T315I substitution introduces a bulky amino acid that causes a major steric clash with (and ensuing insensitivity to) all currently available ATP-competitive TKIs with the sole exception of ponatinib, a multikinase inhibitor with important safety concerns and significant dropout rates among patients, due to side effects. Recently, a novel allosteric inhibitor, asciminib, was developed to provide an additional choice for these patients. As evolutionary cancer dynamics can potentially lead to the selection of a resistant clone for every therapy [12], adding one more drug to the available array would provide better chances to overcome resistance mechanisms. Pexmetinib is well tolerated in patients, however, it shows high pharmacokinetics variability, due to inconsistent absorption [13]. Indeed, we initially attempted oral pexmetinib dosing in mice and only observed 20% tumor inhibition compared to vehicle (data not shown). Nevertheless, mean peak exposure in humans (783 ng/ml, or 1407 nM; Supplementary Table 3) is well above the IC_50_ (411 nM) and near the IC_75_ (1418 nM) obtained with Ba/F3^T315I^ cells.

To date, pexmetinib has been evaluated in phase 1 clinical trials for myelodysplastic syndromes as a monotherapy [13], and in solid tumors in combination with immune checkpoint inhibitors [14]. A bioavailability study was conducted in healthy subjects to optimize pharmaceutical formulation [15].

In conclusion, these results indicate that pexmetinib could be tested in clinical trials for TKI-resistant CML or it may be used as a lead compound for an optimization campaign aimed at improving the pharmacokinetic profile.

### Supplementary information

Supplementary data

## Data Availability

All data generated or analyzed during this study are included in this published article and its Supplementary Information files.
