# Supplementary material for: Repurposing pexmetinib as an inhibitor of TKI-resistant BCR::ABL1
Source: Leukemia. 2024 May 15;38(8):1843–7. doi: 10.1038/s41375-024-02282-y (PMC11286508; doi:10.1038/s41375-024-02282-y)
Supplement: Supplementary file 1 — Supplementary data [file 41375_2024_2282_MOESM1_ESM.docx]

**SUPPLEMENTARY INFORMATION**

- Supplementary Methods
- Supplementary Table 1. IC50s of ABL1 inhibitors on Ba/F3-BCR::ABL1 cells
- Supplementary Table 2. Therapeutic index of ABL1 inhibitors on Ba/F3-BCR::ABL1 cells
- Supplementary Table 3. Pharmacokinetics data of ABL1 inhibitors
- Supplementary Figure 1. Screening strategy
- Supplementary Figure 2. Compounds passing the filter in screening phase 1
- Supplementary Figure 3. Dose-response curves of inhibitors on Ba/F3-BCR::ABL1 and human CML cells
- Supplementary Figure 4. Sensitivity of Y253F and Y253H mutants to pexmetinib
- Supplementary Figure 5. Virtual docking of Y253F and Y253H mutants to pexmetinib
- Supplementary references

**Supplementary Methods**

*Drugs and cell lines*

The Kinase Inhibitor Library (cat# 1200) and pexmetinib (cat# S7799) were purchased from Selleck Chemicals (Houston, TX, USA). The murine pro-B cell line Ba/F3 was purchased from ATCC and cultured in RPMI 1640 supplemented with 10% fetal bovine serum (FBS), 2 mmol/L L-glutamine, 100 units/mL penicillin G, 80 mg/mL gentamicin, and Chinese hamster ovary–conditioned medium as a source of interleukin-3 (IL-3), at 37˚C in 5% CO_2_ atmosphere. Ba/F3 cells expressing WT and mutant BCR::ABL1 were obtained by electroporation as previously described (1). Human Ph+ CML cell line KCL22 and its drug-resistant derivatives were maintained in RPMI 1640 with 10% FBS and antibiotics. KCL-DasR and KCL-BosR were obtained by culturing parental KCL22 in increasing dasatinib and bosutinib concentrations, respectively, until a population was obtained that could grow in 100 nM drug (2). The sequence of BCR::ABL1 kinase domain from drug-resistant cells was determined by reverse transcription of total RNA and Sanger sequencing, as described (1).

*Cell viability and colony assays*

Cells (10^4^/well) were seeded in triplicate in 96-well microplates in the presence of vehicle (DMSO) or compounds for 72 hours. For time course experiments, the cells were followed over 9 days with dilution on day 4 to avoid saturation. Cell growth and viability were assessed using the CellTiter 96® Aqueous One Solution Cell Proliferation Assay System (Promega, Madison, WI, USA) following manufacturer’s instructions. Dose-response curves were built by non-linear fitting of normalized cell growth data using GraphPad Prism software. For colony assays, the cells (3×10^4^ per well) were seeded in 6-well plates in methylcellulose semisolid medium (Methocult H4034; StemCell Technologies, Meda, Italy) in the presence of inhibitors at 37°C, and colonies were counted after 14 days.

*Western blotting*

The cells were seeded in 6-well plates and treated with inhibitors for 4 hours. Equal amounts of total cell lysates were loaded on SDS-PAGE and analyzed as described (3), with phospho-c-Abl (Tyr245) antibody (#2861, Cell Signaling Tech.; diluted 1:1000) and total c-Abl antibody (clone K-12; #sc-131, Santa Cruz Biotech.; diluted 1:500).

*Kinase assay*

A radiometric protein kinase assay was run at Reaction Biology Europe GmbH using purified recombinant ABL1 and ABL1-T315I kinases in the presence of [γ-^33^P]-ATP, poly(Ala,Glu,Lys,Tyr) substrate, and increasing pexmetinib concentrations. Dose-response curves were generated by non-linear fitting of control-normalized data.

*In vivo efficacy*

Ten six-week-old female athymic Nude (Foxn1^nu/nu^) mice were purchased from Envigo (Milan, Italy) and kept under standard conditions following guidelines by the University of Milano-Bicocca ethical committee for animal welfare. The protocol was approved by the Italian Ministry of Health. The mice were injected subcutaneously with 8×10^6^ cells in the right flank. Daily oral (by gavage) or intraperitoneal (i.p.) treatments started when tumors were measurable ( ̴30 mm^3^ average). For oral dosing, the vehicle was 0.5% carboxymethylcellulose/0.1% Tween 80. For i.p. administration, pexmetinib was dissolved in 25% ethanol, 25% Cremophor EL and 50% saline. Tumor volume was measured with a caliper using the formula: Volume (mm^3^) = d^2^ x D/2 where *d* is the shortest and *D* is the longest diameter of the tumor.

*Molecular modelling*

Docking studies were performed using the Maestro suite from Schrodinger (Maestro, version 9.8, Schrödinger, LLC, New York, NY, 2013). We obtained the crystal structures of WT and T315I ABL1 from the Protein Data Bank (rcsb.org). The proteins were prepared using the Protein Preparation Wizard in Maestro and the receptor grid was generated using default settings around the ligand of the original structures. Docking was performed at XP (extra precision) setting and a maximum of 10 docking solutions were stored and re-scored using the strain energy calculation and re-scoring routine of Maestro.

**Supplementary Table 1.** The IC_50_ values obtained in 72h viability assays are reported for all compounds across all Ba/F3 cell lines tested. Data are expressed in nanomolar units (average of three experiments).

| **Ba/F3** | Imatinib | Nilotinib | Dasatinib | Bosutinib | Ponatinib | Rebastinib | Pexmetinib |
| --- | --- | --- | --- | --- | --- | --- | --- |
| **Parental** | 5683 | 680 | 1040 | 1594 | 1197 | 299 | 12238 |
| **WT** | 141 | 5 | 0.4 | 10 | 0.3 | 23 | 31 |
| **T315I** | 9221 | 697 | 1373 | 1890 | 6 | 15 | 411 |
| **G250E** | 3613 | 81 | 8.1 | 179 | 13 | 69 | 196 |
| **Y253F** | 1888 | 57 | 2.9 | 40 | 8 | 52 | 232 |
| **E255K** | 3174 | 118 | 10.3 | 394 | 18 | 81 | 298 |
| **E255V** | 8953 | 182 | 6.3 | 230 | 27 | 48 | 387 |

**Supplementary Table 2.** Therapeutic index of compounds. The IC_50_ ratio (defined here as therapeutic index, as detailed in the formula below) was calculated from the data reported in Supplementary Table 1.

| **Ba/F3** | Imatinib | Nilotinib | Dasatinib | Bosutinib | Ponatinib | Rebastinib | Pexmetinib |
| --- | --- | --- | --- | --- | --- | --- | --- |
| **WT** | 40 | 144 | 2922 | 163 | 3734 | 13 | 395 |
| **T315I** | 1 | 1 | 1 | 1 | 190 | 20 | 30 |
| **G250E** | 2 | 8 | 128 | 9 | 96 | 4 | 62 |
| **Y253F** | 3 | 12 | 359 | 40 | 153 | 6 | 53 |
| **E255K** | 2 | 6 | 101 | 4 | 68 | 4 | 41 |
| **E255V** | 1 | 4 | 165 | 7 | 44 | 6 | 32 |

IC_50_ (*par*)

IC_50_ (*mut*)

therapeutic index^*^ =

**par*, parental Ba/F3 cells+IL3

*mut*, Ba/F3 cells expressing BCR::ABL1 mutants

**Supplementary Table 3.** Pharmacokinetics data of ABL1 inhibitors, extracted from the literature.

| Drug | Dose | C_max_ (nM) | *Reference* |
| --- | --- | --- | --- |
| Pexmetinib | 400 mg qd | 1407 | *Garcia-Manero G, et al.* (4) |
| Imatinib | 400 mg qd | 1594 | *Peng B, et al.*(5) |
| Nilotinib | 300 mg bid | 2568 | *Tian X, et al.* (6) |
| Dasatinib | 70 mg bid | 113 | *Breccia M & Alimena G* (7) |
| Bosutinib | 400 mg qd | 166 | *Abbas R, et al.* (8) |
| Ponatinib | 45 mg qd | 145 | *Cortes JE, et al.* (9) |
| Rebastinib | 150 mg bid | 556 | *Cortes JE, et al.* (10) |

**Supplementary Figure 1**

**A**

phase 1

phase 2

phase 3

627 cpds

27 cpds

3 cpds

pexmetinib

**pass filter**

% viability IL3

% viability T315I

>5

IC_50_ IL3

IC_50_ T315I

>3

**B**


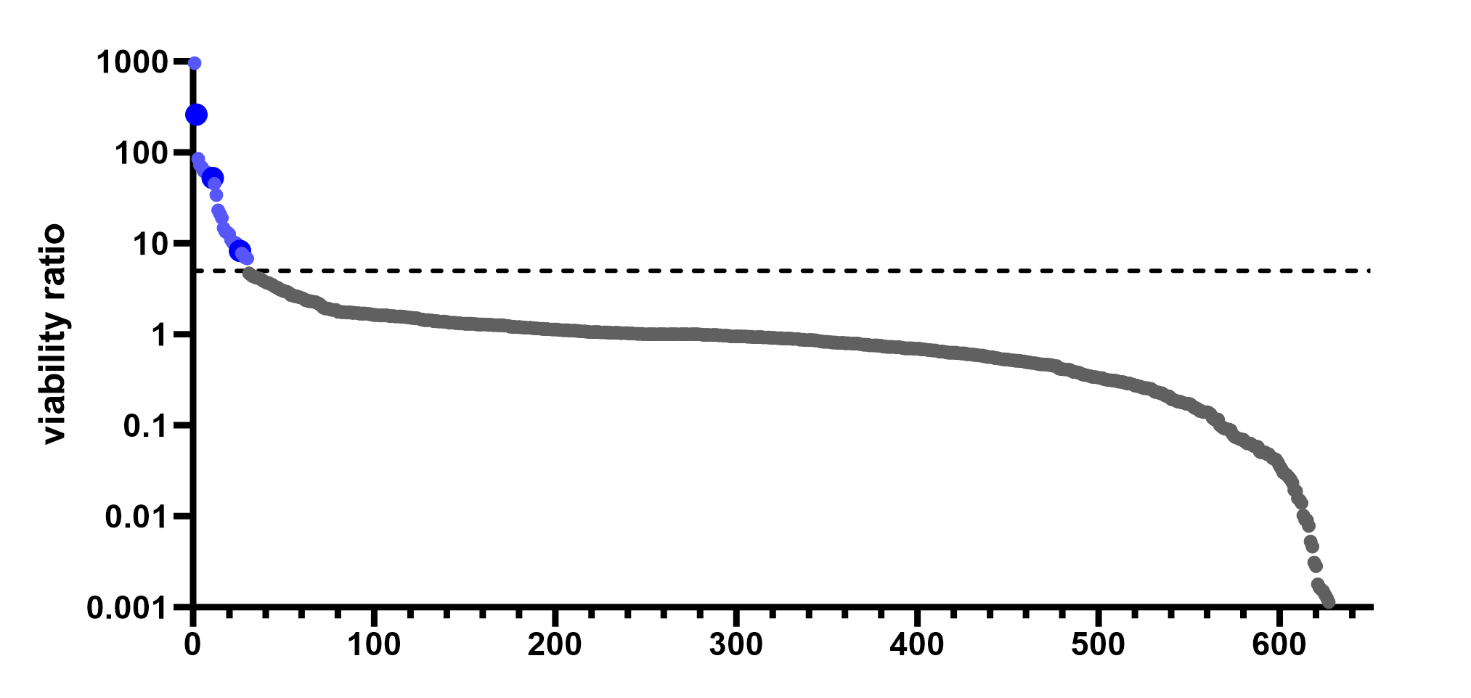


**compounds**

**ponatinib**

**rebastinib**

**pexmetinib**

**Supplementary Figure 1. Compound screening.** (**A**) A commercial compound library containing 627 kinase inhibitors was screened by MTS assay as described in the text. Pass filter criteria are indicated in the figure: a viability ratio >5 in favor of parental Ba/F3 cells to identify selective activity using a single compounds concentration (phase 1); an IC_50_ ratio >3 using dose-response curves in phase 2; phase 3 refers to the detailed characterization of pexmetinib. (**B**) Plot of viability ratio results from all 627 drugs tested in phase 1 of the screening. A dotted line marks the threshold (ratio = 5) to pass phase 1 filter. Compounds passing the screening are shown in blue.

**Supplementary Figure 2**

**Supplementary Figure 2.** List of the 27 compounds passing the threshold in phase 1.

**Supplementary Figure 3**


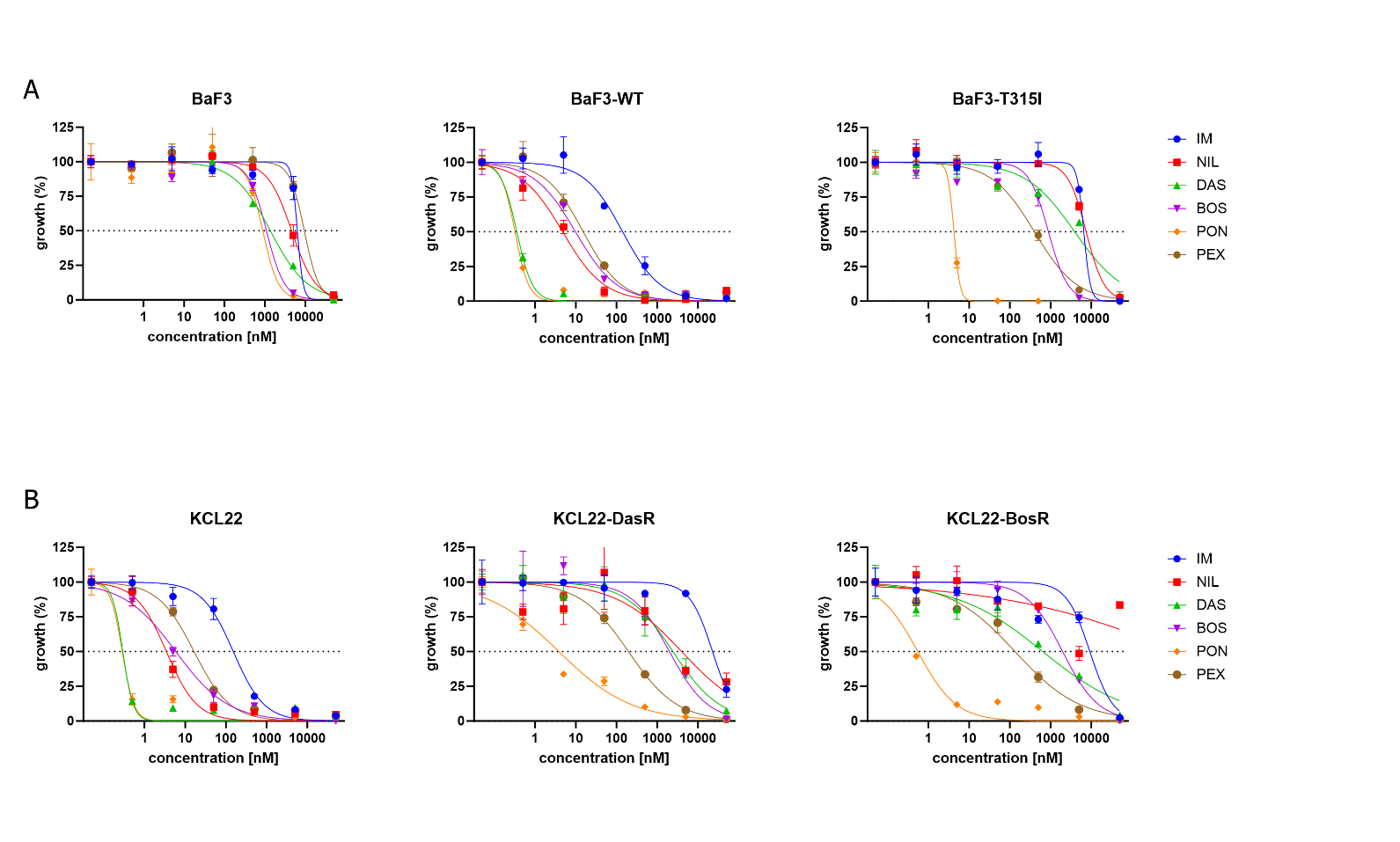


**Supplementary Figure 3.** Ba/F3 cells, parental or expressing the WT or T315I mutant BCR::ABL1 fusion as indicated above graphs (**A**), and human Ph+ CML cells, TKI sensitive (KCL22) or resistant (KCL22-DasR and KCL22-BosR) (**B**), were treated with increasing concentrations of the indicated inhibitors. Non-linear fitting of normalized cell growth is shown. IM=imatinib; NIL=nilotinib; DAS=dasatinib; BOS=bosutinib; PON=ponatinib; PEX=pexmetinib.

**Supplementary Figure 4.**


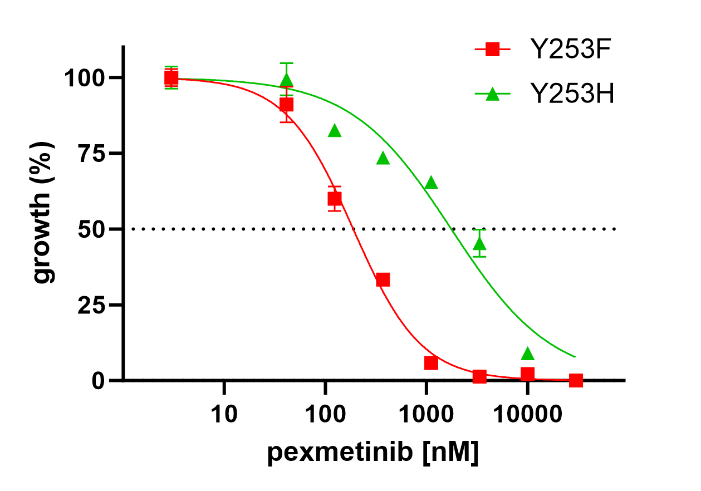


**Supplementary Figure 4.** Ba/F3 cells expressing the Y253F or Y253H BCR::ABL1 mutants were challenged with pexmetinib.

**Supplementary Figure 5.**

**
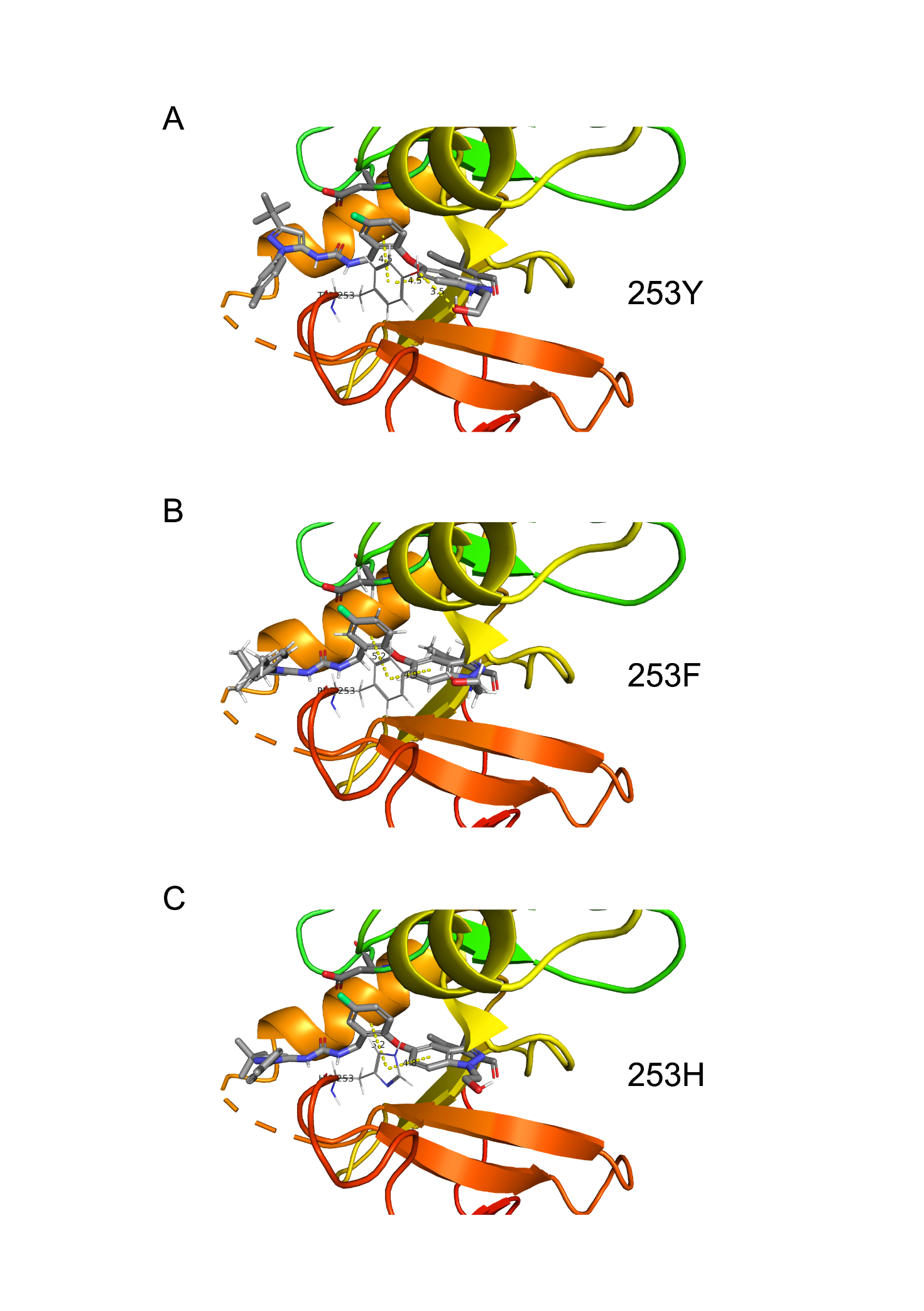
**

**Supplementary Figure 5.** Comparison of the molecular docking of pexmetinib (grey sticks) on ABL1 carrying the normal Tyr253 (PDB: 4TWP) (**A**) and on models of ABL1^Y253F^ (**B**) and ABL1^Y253H^ (**C**).

**Supplementary references**

1. Redaelli S, Mologni L, Rostagno R, Piazza R, Magistroni V, Ceccon M, et al. Three novel patient-derived BCR/ABL mutants show different sensitivity to second and third generation tyrosine kinase inhibitors. Am J Hematol. 2012;87:E125-8.

2. Le Coutre P, Tassi E, Varella-Garcia M, Barni R, Mologni L, Cabrita G, et al. Induction of resistance to the Abelson inhibitor STI571 in human leukemic cells through gene amplification. Blood. 2000;95:1758–66.

3. Arosio G, Sharma GG, Villa M, Mauri M, Crespiatico I, Fontana D, et al. Synergistic Drug Combinations Prevent Resistance in ALK+ Anaplastic Large Cell Lymphoma. Cancers (Basel). Switzerland; 2021;13:4422.

4. Garcia-Manero G, Khoury HJ, Jabbour E, Lancet J, Winski SL, Cable L, et al. A phase I study of oral ARRY-614, a p38 MAPK/Tie2 dual inhibitor, in patients with low or intermediate-1 risk myelodysplastic syndromes. Clin Cancer Res. 2015;21:985–94.

5. Peng B, Hayes M, Resta D, Racine-Poon A, Druker BJ, Talpaz M, et al. Pharmacokinetics and pharmacodynamics of imatinib in a phase I trial with chronic myeloid leukemia patients. J Clin Oncol. 2004;22:935–42.

6. Tian X, Zhang H, Heimbach T, He H, Buchbinder A, Aghoghovbia M, et al. Clinical Pharmacokinetic and Pharmacodynamic Overview of Nilotinib, a Selective Tyrosine Kinase Inhibitor. J Clin Pharmacol. 2018;58:1533–40.

7. Breccia M, Alimena G. Activity and safety of dasatinib as second-line treatment or in newly diagnosed chronic phase chronic myeloid leukemia patients. BioDrugs. 2011;25:147–57.

8. Abbas R, Hug BA, Leister C, Gaaloul M El, Chalon S, Sonnichsen D. A phase I ascending single-dose study of the safety, tolerability, and pharmacokinetics of bosutinib (SKI-606) in healthy adult subjects. Cancer Chemother Pharmacol. 2012;69:221–7.

9. Cortes JE, Kantarjian H, Shah NP, Bixby D, Mauro MJ, Flinn I, et al. Ponatinib in refractory Philadelphia chromosome-positive leukemias. N Engl J Med. 2012;367:2075–88.

10. Cortes J, Talpaz M, Smith HP, Snyder DS, Khoury J, Bhalla KN, et al. Phase 1 dose-finding study of rebastinib (DCC-2036) in patients with relapsed chronic myeloid leukemia and acute myeloid leukemia. Haematologica. 2017;102:519–28.
